# The Seasonal Dynamics and the Influence of Human Activities on Campus Outdoor Microbial Communities

**DOI:** 10.3389/fmicb.2019.01579

**Published:** 2019-07-10

**Authors:** Chaoyun Chen, Ruiqiao He, Zhangyu Cheng, Maozhen Han, Yuguo Zha, Pengshuo Yang, Qi Yao, Hao Zhou, Chaofang Zhong, Kang Ning

**Affiliations:** Key Laboratory of Molecular Biophysics of the Ministry of Education, Hubei Key Laboratory of Bioinformatics and Molecular-Imaging, Department of Bioinformatics and Systems Biology, College of Life Science and Technology, Huazhong University of Science and Technology, Wuhan, China

**Keywords:** campus outdoor microbial communities, seasonal dynamics, human activities, temperature, functional stability

## Abstract

Large-scale campus resembles a small “semi-open community,” harboring disturbances from the exchanges of people and vehicles, wherein stressors such as temperature and population density differ among the ground surfaces of functional partitions. Therefore, it represents a special ecological niche for the study on microbial ecology in the process of urbanization. In this study, we investigated outdoor microbial communities in four campuses in Wuhan, China. We obtained 284 samples from 55 sampling sites over six seasons, as well as their matching climatic and environmental records. The structure of campus outdoor microbial communities which influenced by multiple climatic factors featured seasonality. The dispersal influence of human activities on microbial communities also contributed to this seasonal pattern non-negligibly. However, despite the microbial composition alteration in response to multiple stressors, the overall predicted function of campus outdoor microbial communities remained stable across campuses. The spatial–temporal dynamic patterns on campus outdoor microbial communities and its predicted functions have bridged the gap between microbial and macro-level ecosystems, and provided hints toward a better understanding of the effects of climatic factors and human activities on campus micro-environments.

## Introduction

Large-scale campus, consisting of gates, teaching buildings, school service buildings, living quarters, roads, and other facilities of varying sizes, wherein campus dwellers are exposed to complex macro-environment and micro-environment within a “semi-open community.” Living within a “semi-open community,” students go out of the campus seldom and spend the most time in the campus. Comparing to other working place or entertainment venues as well nature or artificial ecological niche, in the campus, there are almost daily social activities, harboring disturbances from the exchanges of people and vehicles. Such a semi-open community could be roughly defined as a sociological and biological community with constraint access of persons from outside with gates and hotels as the interfaces, which is strongly affected by environmental stressors like temperature (Bahram et al., 2018) and population density (Yan et al., 2016). With the moving persons and vehicles as hosts, so do microbes move around the campuses, following the same routes as persons and vehicles on which they temporarily habit. This make ups the link among certain sets of locations on campus. And the outdoor microbial ecology on campus is a new niche for microbial communities profiling, completely different from the indoor microbial communities that have been examined extensively. And it represents an important niche between large city boroughs (Bik et al., 2016) and a relatively small occupant apartment. The activities of dwellers and vehicles of large-scale campuses could in turn profoundly affect their surrounding environment. However, how spatial and temporal dynamics of campus outdoor microbial communities are affected by human activities remains unclear and there still lack systematic and comprehensive investigations for campus outdoor microbiome.

Microbial ecology plays a significant role in the biogeochemical cycle, as well as the regional nutrient cycle such as microbes in soil (Wall, 2004; Bossio et al., 2005; Barberán et al., 2011), ocean (Wang et al., 2012; Fuhrman et al., 2015), and lake (Li et al., 2015; Han et al., 2016; Ward et al., 2017). As reported, the structure and function of microbial communities vary under urban stress (Concepción et al., 2015; Reese et al., 2016), climate (Barberán et al., 2015), and geomorphology (Wang et al., 2012; de Voogd et al., 2015; Sunagawa et al., 2015). With the development of sequencing technology (Loman and Pallen, 2015), the identification and monitoring of large microbial diversity in diverse biological niches had been possible. And general profiles of the interactions between microbiome and the environmental conditions where it lives have been widely reported, suggesting its potential to influence human health (Hanski et al., 2012; Fujimura et al., 2014). Meanwhile, a longitudinal analysis of microbial interaction between humans and the indoor environments indicated that human effects can largely shape the microbial pattern in house surface of occupants (Kembel et al., 2014; Lax et al., 2014; Ruizcalderon et al., 2016; Savage et al., 2016). Similarly, microbial pattern could be influenced by population density within boroughs (Bik et al., 2016).

**FIGURE 1:**
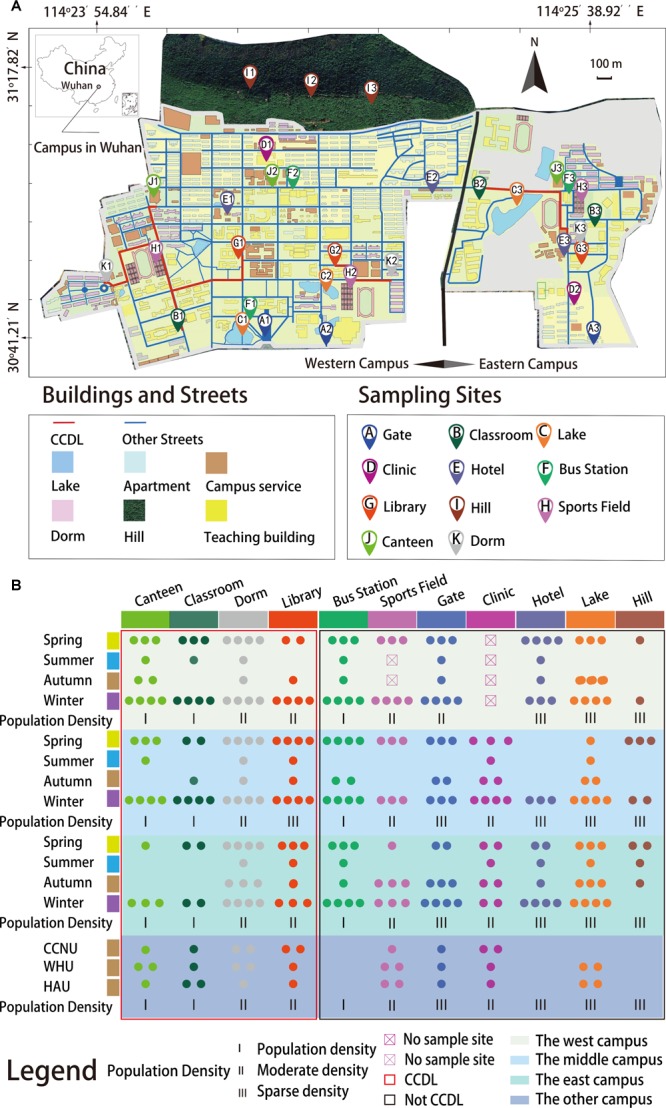
Geological locations and sampling times for the whole sample collection process. **(A)** Samples were collected from 32 sampling sites and marked in the map (samples from other universities were not shown in the map but in **B**). **(B)** Samples were categorized by their sampling times and sites. Actual numbers of samples were represented by the numbers of solid circles in different colors. All samples were collected as planned (refers to the section “Materials and Methods”), while some of the data were filtered out due to low sequencing quality. There were 284 samples representing six seasons for this study.

Considering the potential influences of microbiome on the macro-environment (Falkowski et al., 2008) and human health (Barberán et al., 2015), to understand their interactions in different niches is crucial, as a semi-open community against exchanges of people and vehicles from outside with gates, and hotels as the interface, large-scale campus stands for an important niche between large city boroughs (Reese et al., 2016) and occupant apartments, is a special-ecological niche for the study on microbial ecology in the process of urbanization. Previous study has revealed building-specific and temporal stable pattern of campus indoor bacterial communities (Ross and Neufeld, 2015); however, the outdoor microbial ecology on campus has been rarely studied up till now. Focusing on this unique niche of microbial ecology, a representative large-scale campus, Huazhong University of Science and Technology (HUST), along with other three campuses [Central China Normal University (CCNU), Huazhong Agriculture University (HZAU), and Wuhan University (WHU)] in Wuhan, China, were selected, since they have similar greenery coverage and landscape distribution: artificial or nature lakes, small hills, as well as the arrangement of typical campus buildings (Figure 1A).

The spatial and temporal microbial dynamics of campus outdoor microbial communities of the four campuses and their potential environmental drivers were comprehensively delineated. For this study, a first attempt of this initiative, we have examined the surface microbial communities on campus, obtaining 284 samples from 11 types of sites across seasons (Figure 1B and Supplementary Dataset 1). And we mainly focused on the following questions: (i) Does campus outdoor microbial communities exert seasonal alteration and how? Seasonal factor and other climatic factors were integrated to investigate microbial communities’ chronological dynamics. (ii) Does the human activities influence the microbial communities? Samples were collected according to different sites in HUST (Figure 1A): first, Classroom, Canteen, Dorm, and Library were classified as “CCDL” which routinely accommodates a densely populated flow from the east to the west of the campus. Second, Bus Stations, Sports Fields, Clinics, Gates, and Hotels were typical sites with non-routine human mobility on campus. Third, samples from Hills and Lakes were categorized in another group featuring sparse population in most of the time. Comparisons of the microbial composition in the three groups were applied to assess the effect of human activities. (iii) Does campus outdoor microbial communities feature functional robustness? Cross-campus comparisons of community taxonomical and functional compositions (refers to the Supplementary Information) have been conducted toward this aim. Together, these analyses have offered us a unique lens toward the landscape of campus outdoor microbial communities and its interactions with environmental factors and human activities.

## Results

### General Profile of Campus Outdoor Microbial Communities

**FIGURE 2:**
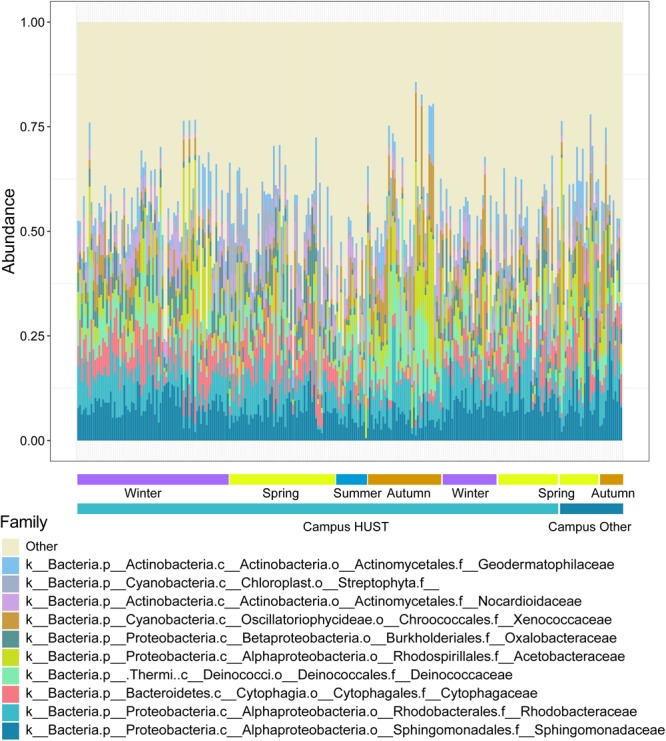
Relative abundances (RAs) of species for 284 samples at family level. The samples were collected from 55 different sites over six seasons from the winter of 2015 to the spring of 2017. RA of top 10 families was depicted by different colors, while taxons with lower RA were classified as “Other.” The horizontal bars at the bottom represented seasonal grouping of samples.

We have generated taxonomical structure for each sample quantitatively, in which the relative abundance (RA) of each species was obtained at family level (Figure 2 and Supplementary Dataset 2). With adequate depth of 16S rRNA gene sequencing (Supplementary Figure 1), 31,523,358 high-quality reads in total for 284 samples (110,998 reads for each sample on average) were obtained after quality control. Totally 541,981 operational taxonomic units (OTUs) were then assigned. The pattern of microbial RA within each season showed comparable homogeneity [PERMANOVA test, *R*^2^ = 0.199, Pr (>*F*) = 0.001]. The dominant phylum for campus outdoor microbial communities include *Proteobacteria, Actinobacteria*, and *Thermi* among all four seasons. While the RA of some families represented their dominance in the bacterial community in certain season, such as *Acetobacteraceae* (average RA = 0.093) generally known as acetic acid bacteria, which was enriched in autumn.

Such community structures have similarities to certain degrees with those found in urban soil at Manhattan (Reese et al., 2016), as the dominant species (top three phylum, *Proteobacteria, Actinobacteria*, and *Bacteroidetes*) resemble those in urban soil at Manhattan (top three phylum, *Proteobacteria, Acidobacteria*, and *Bacteroidetes*) (Reese et al., 2016). But campus outdoor microbial community patterns differ from those found in forest soil (top three phylum, *Proteobacteria, Acidobacteria*, and *Actinobacteria*) (Parks and Beiko, 2010) and those in human gut (gut microbiome, *Firmicutes* over 80% dominance) (Ren et al., 2017). On the other hand, campus outdoor microbial communities also feature characteristic phylum compared to those found in urban soil at Manhattan: *Actinobacteria* were present as one of the dominant phylum, which was not a dominant one in urban soil at Manhattan.

### Seasonality of Campus Outdoor Microbial Communities

**FIGURE 3:**
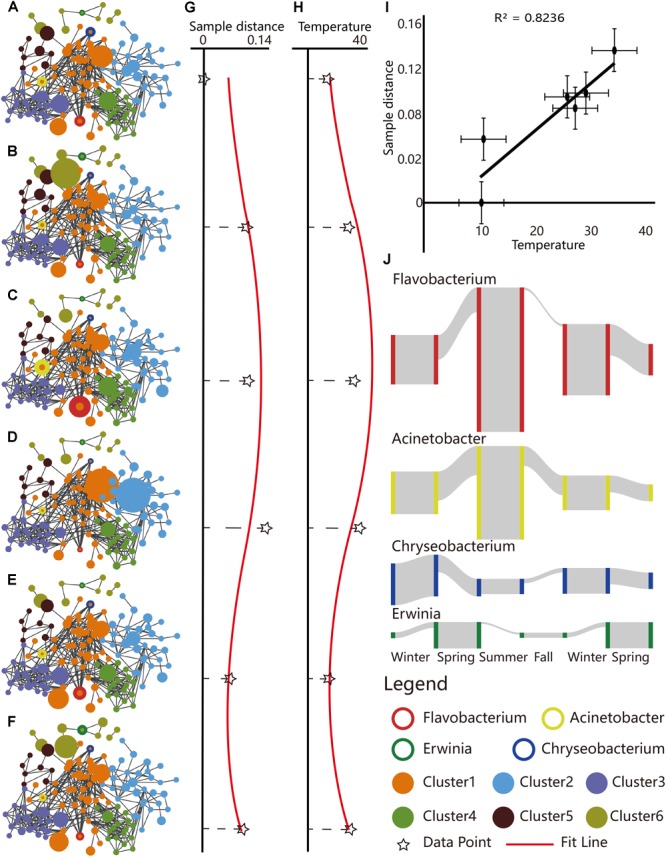
Seasonal dynamics of taxonomical structures reflected by the species–species networks, correlation of sample similarities with temperature, and selected seasonal biomarkers. **(A–F)** Species–species networks were generated based on Spearman correlation similarity matrix at genus level, for total 284 samples collected from **(A)** winter of 2015, **(B)** spring of 2016, **(C)** summer of 2016, **(D)** autumn of 2016, **(E)** winter of 2016, and **(F)** spring of 2017. The sizes of the circles represented the RA of OTUs for a certain season. Scatter plots presented sample similarities of **(G)** microbial community composition and **(H)** average temperature during the six seasons. The RA table of microbial community in 2015 winter at genus level served as the reference. The black star marks represented the observed values of sample similarities, while the red curves represented the fitted line of sine function. **(I)** Scatter plot depicted microbial sample similarities of the six seasons versus temperature, which featured a positive linear correlation (Pearson correlation coefficient, *R*^2^ = 0.8236). **(J)** Seasonality of typical biomarkers. The height of the gray column indicated the RA of the corresponding species.

Campus outdoor microbial communities showed seasonal variations at genus level. Species–species networks were constructed at genus level to infer the inter-species co-occurrences within microbial community (Figure 3A–F). The integral pattern of campus outdoor microbial communities showed detectable difference among seasons by PERMANOVA test [*R*^2^ = 0.199, Pr (>*F*) = 0.001]. The sample similarities calculated by Euclidean distance (refers to the Supplementary Information) (Schnorr et al., 2014), based on taxonomical structures among six seasons, performed seasonal alteration (Figure 3G) with fitted curve of the sine function (*T*-test, *p* = 0.191).

Interestingly, average temperature among different sampling sites also featured cyclical variation (*T*-test, *p* = 0.109, Figure 3H), which was in concordance with seasonal sample similarities (Pearson correlation coefficient, *R*^2^ = 0.824, Figure 3I).

To obtain a better understanding of the potential drivers of the campus outdoor microbial communities’ seasonality, we examined the correlations between sample similarities and other climatic factors, including barometric pressure, humidity, and UV index. The barometric pressure in Wuhan, China, presented detectable cyclicality through seasons (Supplementary Figure 2B and Supplementary Dataset 3), negatively correlated to sample similarities (Pearson correlation coefficient, *R*^2^ = 0.666, Supplementary Figure 2D). Average UV index showed seasonality (Supplementary Figure 3A) and was positively correlated to sample similarities (*R*^2^ = 0.115, Supplementary Figure 3B). However, there was no obvious cyclical behavior of average humidity in Wuhan (Supplementary Figure 3C), and the correlation between humidity and sample similarities over seasons was weak (Supplementary Figure 3D).

Some species exerted their distinguished RA in a certain season, serving as a potential seasonal biomarker (Supplementary Figure 4A). For example, *Erwinia* (LEfSe, LDA = 3.964, *p* = 2.07*e*-19, plant pathogenic species) was enriched in spring, *Flavobacterium* (LDA = 4.681, *p* = 3.46*e*-24, freshwater fish pathogen), and *Acinetobacter* (LDA = 4.364, *p* = 7.80*e*-07, soil mineralization) were the two for summer, while *Chryseobacterium* (LDA = 4.062, *p* = 9.65*e*-14, cold tolerance) was found to indicate the duration of winter (Figure 3J).

### Human Activities Influence Campus Outdoor Microbial Communities

On campus, roads connecting CCDL accommodate a routinely large flux of students, while other roads feature sparse (roads connecting Hills and Lakes) or non-routine (roads connecting Clinics, Gates, Hotels, Bus Station, and Sports Fields) human activities (Figure 1A). The comparison between these three groups could shed light on the influence of human activities on campus outdoor microbial communities.

**FIGURE 4:**
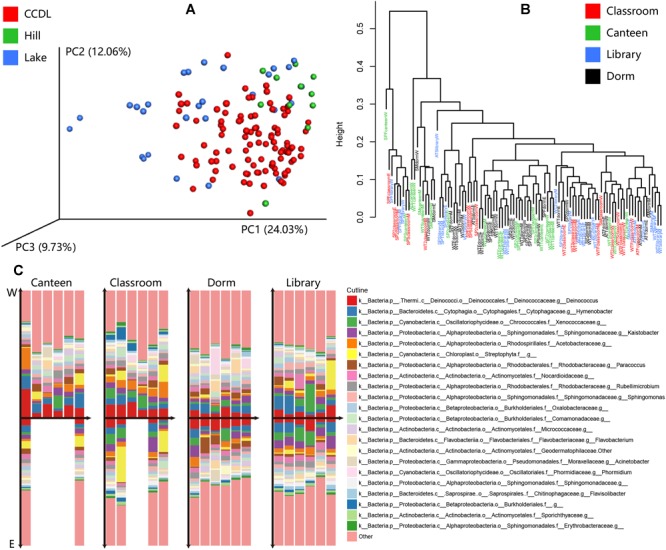
Taxonomical composition of microbial communities at family level between CCDL and others, within CCDL, and between eastern and mid-western parts of campus. **(A)** Principal coordinates analysis (PCoA) for the microbial samples from CCDL, Lake, and Hill based on Weighted Unifrac distance. **(B)** Hierarchical clustering of samples among CCDL: canteen, classroom, dorm, and library based on Euclidean distance. **(C)** Compositional differences of samples collected on the ground surface of canteen, classroom, dorm, and library, between eastern campus (refers to “E”) and mid-western campus (middle and west parts of the HUST campus, refers to “W”). In each group, columns were arrayed according to season (from winter of 2015 to spring of 2017), with the above part elucidating data from the mid-western campus and those below from eastern campus.

The intensity and variety of activities together influenced the compositional pattern of campus outdoor microbial communities. Principal coordinate analysis (PCoA) and PERMANOVA test of the microbial communities’ composition showed tremendously distinction among CCDL, Hill, and Lake [PERMANOVA test, *R*^2^ = 0.129, Pr (>*F*) = 0.001, Figure 4A]. And the result of heatmap in Supplementary Figure 5A also indicated this distinction. The gradual shift of microbial taxonomical composition from CCDL to Hill and Lake might explain the dispersal influence of human activities on campus outdoor microbial communities, since Lake was in the vicinity of CCDL compared to Hill.

The varieties of activities differ among CCDL, the microbial taxonomical compositions of CCDL, however, showed no detectable difference (Figure 4B). To explore if geographic distribution of sampling sites influenced the comparison, the taxonomic structures of the microbial communities between eastern and mid-western of each CCDL (Supplementary Figure 5B) were compared, the microbial communities’ compositional pattern of which turned out to perform no marked differences [PERMANOVA test, *R*^2^ = 0.031, Pr (>*F*) = 0.211, Figure 4C].

To further investigate the effects of human activities on campus outdoor microbial communities, and to exclude the effects of measured temperature as a confounding factor, we have further evaluated the association of microbial taxonomical compositions with human activities, one season at a time. Results have shown that distinction are very clear among CCDL, Hill, and Lake [PERMANOVA test, *R*^2^ = 0.2655, Pr (>*F*) = 0.001 for spring; PERMANOVA test, *R*^2^ = 0.236, Pr (>*F*) = 0.001 for winter], verifying the profound influence of human activities on campus microbial communities. Besides, the sports fields, which carries a lot of students and staffs, is a site with special human activities. Significant distinction of microbial community between sports field and other sampling sites [PERMANOVA test, *R*^2^ = 0.134, Pr (>*F*) = 0.001, in general and PERMANOVA test, *R*^2^ = 0.231, Pr (>*F*) = 0.0001 for spring; PERMANOVA test, *R*^2^ = 0.199, Pr (>*F*) = 0.0001 for winter; ABD PERMANOVA test, *R*^2^ = 0.175, Pr (>*F*) = 0.0007 for autumn] had been found also. Proving that the human activities in the sports field influence the composition pattern of the campus outdoor microbial communities. In addition, the PERMANOVA test results (Supplementary Table 1) show that the gates [*R*^2^ = 0.0167, Pr (>*F*) = 0.002, in general], the hotels [*R*^2^ = 0.0128, Pr (>*F*) = 0.007, in general], the dorms [*R*^2^ = 0.0176, Pr (>*F*) = 0.0008, in general], the lakes [*R*^2^ = 0.032, Pr (>*F*) = 0.0001, in general; *R*^2^ = 0.053, Pr (>*F*) = 0.0014, in winter; and *R*^2^ = 0.047, Pr (>*F*) = 0.0066, in spring], the stations [*R*^2^ = 0.012, Pr (>*F*) = 0.009, in general], the clinics [*R*^2^ = 0.022, Pr (>*F*) = 0.001, in general], and the hills [*R*^2^ = 0.013, Pr (>*F*) = 0.009, in general; *R*^2^ = 0.047, Pr (>*F*) = 0.009, in winter] also hold special microbial community structure. These statistical results have supported the hypothesis that human activities can affect the campus outdoor microbial communities.

### Functional Stability Against Human Activities

Campus outdoor microbial communities performed functional stability against multiple stressors. For functional profiling, based on the present acquired 16S rRNA gene sequencing data, the various kinds of predictable methods like PICRUSt (Langille et al., 2013), Tax4Fun (Aßhauer et al., 2015), and FAPROTAX (Louca et al., 2016) can be the only option for microbial functional profiling. Here the results of PICRUSt have been shown, supplemented by the results of Tax4Fun and FAROTAX.

**FIGURE 5:**
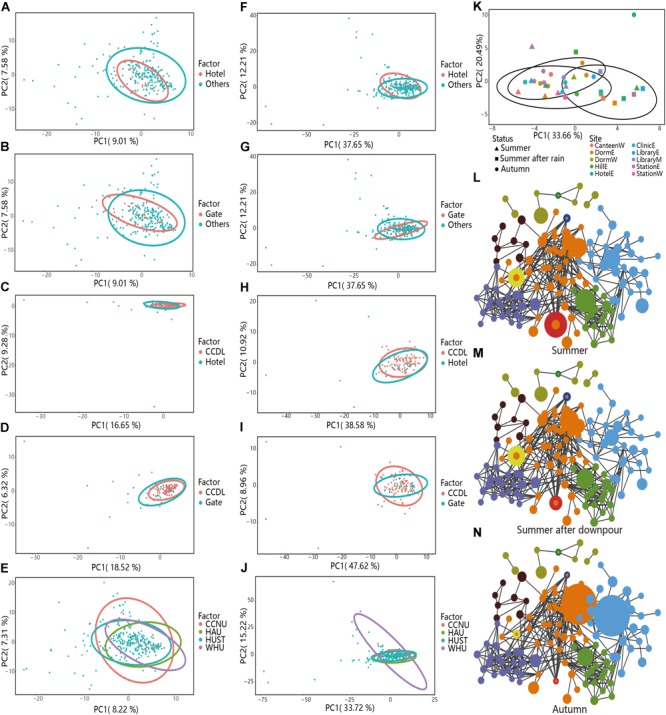
Taxonomical and functional stability assessment of campus outdoor microbial community through PCA and co-occurrence network analysis. **(A–E)** PCA analysis of the taxonomical compositions in different groups. For “Hotels and Others” and “Gate and Others,” 251 samples in HUST were analyzed at family level. As to “CCDL and Hotels,” 121 samples in HUST were included for analysis at family level, and for “CCDL and Gates,” 124 samples in HUST were analyzed at family level. For the comparison of microbial communities from different universities, totally 284 samples were collected from HUST, HZAU, CCNU, and WHU. **(F–J)** PCA was applied for comparison of samples’ functional structures in different groups. **(K)** PCA of the taxonomical compositions in samples collected in HUST in the summer of 2016 (10 samples), after the summer downpour (10 samples), and in the autumn of 2016 (14 samples). Samples from different sites were colored differently, while the shape of the scatters indicated three different sampling times with corresponding confidence ellipses. Co-occurrence networks were also generated to reveal the overall structure dynamics of microbial communities **(L)** in the summer of 2016, **(M)** after the summer downpour of 2016, and **(N)** in the autumn of 2016.

The table including the predicted functions by PICRUSt was provided in Supplementary Dataset 4. Gates and hotels were regarded as the interface between inside and outside of campus, harboring disturbances from the exchanges of people and vehicles. Principal component analysis (PCA) demonstrated relative taxonomic difference between Hotels [PERMANOVA test, *R*^2^ = 0.013, Pr (>*F*) = 0.007, Figure 5A] or Gates [PERMANOVA test, *R*^2^ = 0.017, Pr (>*F*) = 0.001, Figure 5B] versus others. However, for predicted function, the two comparisons showed coherent convergence [Hotels versus Others, PERMANOVA test, *R*^2^ = 0.002, Pr (>*F*) = 0.598, Figure 5F; Gates versus Others, *R*^2^ = 0.006, Pr (>*F*) = 0.187, Figure 5G]. The high similarity in functional composition among different partitions demonstrated functional stability of campus outdoor microbial communities in response to human activities.

To verify the functional robustness of campus microbial communities as identified by PICRUSt, we have also applied Tax4Fun and FAPROTAX analyses on the same sets of samples. Results have shown that as regard to taxonomical composition, distinctions between samples from different sampling sites were clear (Hotels versus Others, PERMANOVA test, *R*^2^ = 0.013, Pr (>*F*) = 0.007, Figure 5A), Gates versus Others, PERMANOVA test, *R*^2^ = 0.017, Pr (>*F*) = 0.001, Figure 5B). While as regard to functional compositions, differences between samples from different sampling sites were not as distinct (Hotels versus Others, PERMANOVA test, *R*^2^ = 0.002, Pr (>*F*) = 0.598, Supplementary Table 1; Gates versus Others, PERMANOVA test, *R*^2^ = 0.006, Pr (>*F*) = 0.19, Supplementary Table 1), indicating the stronger robustness at functional level. And the results based on FAPROTAX have also confirmed functional robustness [Hotels versus Others, PERMANOVA test, *R*^2^ = 0.001, Pr (>*F*) = 0.901, Supplementary Table 1, Gates versus Others, PERMANOVA test, *R*^2^ = 0.004, Pr (>*F*) = 0.356, Supplementary Table 1].

Then we focused on contrasting the taxonomical and functional compositions of CCDL to those of Gates or Hotels. After curtailing sample range from all other sites (except for Gates and Hotels) to CCDL, we noticed a disparate distribution of microbial communities’ composition between CCDL and Hotels [PERMANOVA test, *R*^2^ = 0.032, Pr (>*F*) = 0.002, Figure 5C] or Gates [*R*^2^ = 0.043, Pr (>*F*) = 0.0002, Figure 5D]. The functional analysis of the two comparisons showed no significant difference [CCDL versus Hotels, PERMANOVA test, *R*^2^ = 0.004, Pr (>*F*) = 0.627, Figure 5H; CCDL versus Gates, *R*^2^ = 0.008, Pr (>*F*) = 0.330, Figure 5I]. Results based on Tax4Fun analysis have also shown strong robustness as regard to functional composition [CCDL versus Hotels, PERMANOVA test, *R*^2^ = 0.016, Pr (>*F*) = 0.096, Supplementary Table 1; CCDL versus Gates, *R*^2^ = 0.039, Pr (>*F*) = 0.0014, Supplementary Table 1]. And the results based on FAPROTAX have also confirmed functional robustness [CCDL versus Hotels, PERMANOVA test, *R*^2^ = 0.002, Pr (>*F*) = 0.872, Supplementary Table 1, CCDL versus Gates, PERMANOVA test, *R*^2^ = 0.003, Pr (>*F*) = 0.770, Supplementary Table 1].

Based on the above results, we speculated that the taxonomical structures of campus outdoor microbial communities might change in response to variable stressors such as temperature, barometric pressure, and human activities. However, their functional performance remained considerably robust against the environment (Bossio et al., 2005; Chaffron et al., 2010) and human (Lax et al., 2014) factors. This indicated that the campus outdoor microbial communities might vary their taxonomical abundance to cope with outer stressors on the one hand, and recruit different sets of species to sustain its functional stability on the other hand. This was in accordance with the results of microbial ecology in multiple environments (Rosenfeld, 2002; Taxis et al., 2015).

To evaluate the prevalence of the microbial pattern, we further examined the taxonomical and functional composition of microbial communities from the other three campuses CCNU, HAU, and WHU in Wuhan, China. Comparable patterns of microbial compositions among samples from HUST, CCNU, HAU, and WHU were also characterized, featuring a random distribution of taxonomic composition [PERMANOVA test, *R*^2^ = 0.025, Pr (>*F*) = 0.003, Figure 5E]. The coherence of functional composition among the four universities was also significant [PERMANOVA test, *R*^2^ = 0.008, Pr (>*F*) = 0.478, with the PICRUSt-predicted functions, Figure 5J], and *R*^2^ = 0.009, Pr (>*F*) = 0.420 with FAPROTAX-predicted functions; *R*^2^ = 0.017, Pr (>*F*) = 0.076 with Tax4Fun-predicted functions, indicating the prevalence of functional stability of campus outdoor microbial communities. More details about these PERMANOVA tests based on PICRUSt functional annotations were provided in Supplementary Table 1.

Furthermore, this pattern of functional stability was corroborated by PERMANOVA test based on functions predicted by FAPROTAX (Louca et al., 2016). The differences of FAPROTAX functional patterns (data in Supplementary Dataset 5) were insignificant between Hotels and Others [PERMANOVA test, *R*^2^ = 0.001, Pr (>*F*) = 0.901], CCDL and Gates [PERMANOVA test, *R*^2^ = 0.003, Pr (>*F*) = 0.770], CCDL and Hotels [PERMANOVA test, *R*^2^ = 0.002, Pr (>*F*) = 0.872], Gates and Others [PERMANOVA test, *R*^2^ = 0.004, Pr (>*F*) = 0.355], and among four campuses [PERMANOVA test, *R*^2^ = 0.009, Pr (>*F*) = 0.420], with the only exception which is between lakes versus others [PERMANOVA test, *R*^2^ = 0.027, Pr (>*F*) = 0.002] and sports fields versus others [PERMANOVA test, *R*^2^ = 0.086, Pr (>*F*) = 0.0001, in general; *R*^2^ = 0.088, Pr (>*F*) = 0.004, in winter; *R*^2^ = 0.259, Pr (>*F*) = 0.0001, in spring]. Possible explanation for this might be that the stability of functional compositions of the campus outdoor microbial communities was perturbed most significantly at the sports fields and the lakes. For the sports fields, the differences are largely due to the fact that the ground surfaces are straightly under the sunshine and rain, as well as exposed to constant incoming and outgoing persons for exercises. However, for the lakes, there was no significant difference detected when test one season a time [PICRUSt-predicted functions, *R*^2^ = 0.022, Pr (>*F*) = 0.103, in winter; *R*^2^ = 0.021, Pr (>*F*) = 0.156, in spring; *R*^2^ = 0.013, Pr (>*F*) = 0.190, in summer; and *R*^2^ = 0.031, Pr (>*F*) = 0.275, in autumn]. It means the functional composition variances are largely due to the seasonal factors rather than human activities.

Interestingly, the functional robustness could be interrupted by drastic changes of campus environment such as the summer downpour of 2016 (Savage et al., 2016). Although the microbial communities’ function endured a sharp shift immediately after the downpour (<24 h) [PERMANOVA test, *R*^2^ = 0.3793, Pr (>*F*) = 0.001], along with microbial community structure variation (Figure 5K) and a typical RA variation of seasonal biomarker (e.g., *Flavobacterium*, Figure 5L–N), this interrupted pattern could be reverted back to the seasonal sine pattern (Figure 3G).

We admit that the robustness of functions of microbial communities that we have observed might not be refined, due to the nature that all functions are predicted based on 16S rRNA. However, these results have provided valuable hints for future examination of microbial communities in semi-open community: as such functional robustness of microbial communities against seasonal changes are of ecological considerable for better understanding of campus microbial communities, we deem further investigation based on metagenomic, metatranscriptomic, and metaproteomic data would be invaluable.

### Multiple Drivers of the Microbial Structure

**FIGURE 6:**
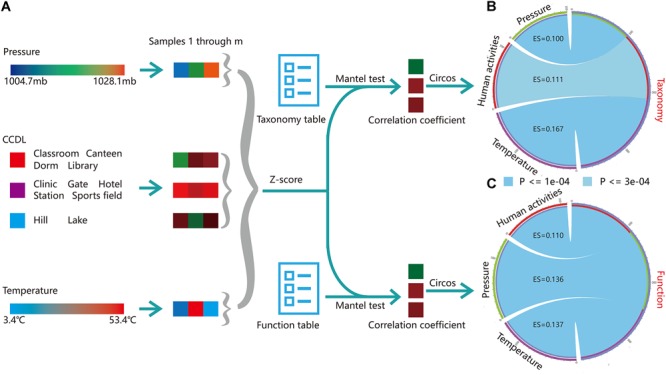
Multi-factor analysis of microbial taxonomical and functional composition. **(A)** Mantel’s test was applied to reveal the correlation of taxonomical (“Taxonomy” in figure) or functional composition (“Function” in figure) versus environmental factors, including barometric pressure (“Pressure” in figure), CCDL, temperature. For CCDL factor, samples from classroom, canteen, dorm, and library were categorized as one group; others were categorized into another group, except for samples from hills and lakes, which were categorized as the third group. Then the environmental information for the three groups was standardized by *Z*-score. Taxonomy table (Supplementary Dataset 7) and function table (Supplementary Dataset 4) along with group information (Supplementary Dataset 1) were prepared for Mantel’s test. **(B)** Visualization of the correlation of taxonomy with barometric pressure, CCDL, and temperature. The arc length of the circle indicated the correlation size. **(C)** The correlation of functional composition with barometric pressure, temperature, and CCDL was obtained similarly.

Campus outdoor microbial communities are exposed to human effects and environmental stressors (e.g., temperature), as a relatively enclosed community. We conducted Mantel’s test to assess the effect size (ES) of these potential drivers on the microbial taxonomy and function among three typical groups [temperature, barometric pressure, and human density, PERMANOVA test, Pr (>*F*) = 0.001, Figure 6A]. Temperature (Mantel’s test, ES = 0.176, *p* = 1*e*-04, Supplementary Figure 5B) influenced profoundly on microbial taxonomy, while human effects (ES = 0.111, *p* = 3*e*-04, Supplementary Figure 5B) and barometric pressure exerted fewer contributions (ES = 0.100, *p* = 1*e*-04, Figure 6B). As for microbial functions, the influences of barometric pressure (Mantel’s test, ES = 0.136, *p* = 1*e*-04) and temperature (ES = 0.137, *p* = 1*e*-04) were stronger than that of human effects (ES = 0.110, *p* = 1*e*-04, Figure 6C). Therefore, we speculated that though campus outdoor microbial communities were under considerable impact from human activities, the climatic factors together would exert stronger influences on the microbial community.

## Discussion

Dozens of studies over the past decade continued to characterize patterns, associations, and drivers of microbial community structures in various built environments, elucidating similarities and differences of the microbial communities in different types of built environments (Mcknight, 2013) and depict the relationships between the human being and the indoor microbes (Sharma et al., 2019) as well as outdoor microbes (Leung and Lee, 2016). However, how spatial and temporal dynamics of campus outdoor microbial communities are affected by human activities and environmental stressors remains unclear, and there still lack systematic and comprehensive researches for campus outdoor microbiome.

In this study, we have expanded the current research on microbiome in built environment to the investigation of a large-scale campus, which represented a special ecological niche for the study on microbial ecology in the process of urbanization. Although campus outdoor microbial communities were exposed to multiple stressors, we primarily focused on their associations with geographical (location), human (human density), and climatic (temperature, barometric pressure, humidity, and UV index) stressors, at taxonomical and functional level. Spatial samples were categorized in terms of sampling sites for investigating the effect of geographical and human stressors, while temporal samples were classified according to seasons to study the dynamics of microbial community responding to the seasonal variation of climatic stressors.

The microbial community pattern of campus outdoor microbial community featured seasonality, influenced by combinations of climatic and human stressors, mainly including temperature and barometric pressure and human density in this study. Seasonal variation of climatic stressors like temperature (Figure 3H and Supplementary Figure 2A) and pressure (Supplementary Figure 2B) is closely related to microbial community’s taxonomical composition. And temperature exerted most contribution to the seasonal changes of taxonomical composition of campus outdoor microbial community (Figure 6). These conclusions were also put forward in previous study in micro environment microbial studies claiming that the temperature and relative humidity influenced the seasonal variations of the indoor microbial communities (Frankel et al., 2012). The human influence on microbial communities of built environments has also been demonstrated in another study on frequently contacted door handles on a university campus which holds high variances (Ross and Neufeld, 2015).

Interestingly, such influence of temperature on microbial community dynamics seems to be ubiquitous. For example, recent studies at global scale have shown that the temperature variation can result in microbial community dynamic changes in the ocean (Klindworth et al., 2014; Fuhrman et al., 2015; Sunagawa et al., 2015). Similar influences could also be found for the forest microbiome (Peñuelas et al., 2012): high temperature or desiccation tends to induce lower microbial richness, resulting in dynamic changes in microbial community in the forest. The equally important stressor on campus outdoor microbial communities is the dispersal influence of human activities, which was in accordance with those reported in the researches at department (Lax et al., 2014), city (Concepción et al., 2015; Reese et al., 2016; Tong et al., 2017), and continent (Barberán et al., 2015) scale. On the other hand, such ubiquity is also site-specific: the microbial functions as well as taxonomical composition of the microbial communities of some places, like sports fields and lakes, were drastically different from other places on the campus. Comparing to other places on the campus, the sports fields exposed straightly to the sunshine, holding amount of intense light every day, which would be a explain of its specialty in the microbial taxonomical structure and the functions. Additionally, as the vicinity of the routine and no-routine students activates in the campus, the microbial functions of lakes also present heterogeneity to other places on the campus.

Comparatively, the geographic stressors wielded less power to engender the spatial difference of microbial communities’ taxonomical composition on campus. The less power of the geographic factors could be explained by the unique feature of the large-scale campus as a semi-open community, on which students and other residents holding repetitive tracks routinely affect the campus outdoor microbial communities. Samples collected on the ground surface of different partitions could largely be relocated and translocated by footprints; therefore, this frequent human mobility in campus could counterbalance the effect of geographic stressors. This speculation of human influence was supported strongly by statistical test: human density explained more of the sample differentiation than the location of the sampling sites did [PERMANOVA test with Pr (>*F*) = 0.001]. And such influences were also exemplified in another setting: recent studies on indoor microbial communities have shown that human beings can be the medium to formulate the microbial signature of house (Lax et al., 2014). The influence of human activities on environmental microbial communities could also be found when monitoring the development of urbanization: the habitat fragmentation produced lots of micro-ecosystem that hold different living conditions for microbes, such as pH, temperature, and urbanization process, leaving a non-negligible impact on the bacterial community of urban soil, exemplified in Beijing city (Yan et al., 2016). Besides, humans and other animal occupants of large-scale campus also have extensive microbial interactions with the air and surfaces. These interactions have traditionally been examined only with regard to the transmission of potential pathogens, yet recent works have revealed more complex role of these microbes. Along this line, it could not prevent us to wonder how to investigate the mechanism behind the impact of human activities on microbial signature in semi-open society like campus as well as the city, for which extensive sampling and deeper data mining would be in urgent need. In summary, new understanding of the microbiology of the built environment and human health has shifted our perspective of microorganisms from a purely negative role (that is, being pathogenic or infectious) to a potentially positive role (that is, protective or preventive). Therefore, it stands to reason that the interactions between humans and environment that facilitate microbial exposure will have a profound impact on human health.

Despite the above stressors altering the microbial taxonomical composition, the functions of campus outdoor microbial communities remained relatively robust. It was noteworthy that the functional stability of campus outdoor microbial communities was most significant against human activities and this pattern was persistent among different campuses in Wuhan, China. To understand this, we should look at it from two sides: on taxonomical level, certain species in the campus outdoor microbial communities would not be adaptive compared to their neighbors, rendering their RA in the community to reduce (Bossio et al., 2005), leading to detectable changes of taxonomical structures of the microbial communities (Sharp et al., 2017; Zhu et al., 2017). On functional level, such drastic changes are not present: previous studies have shown that under constant environmental conditions, the activities of microbial functional groups are not sensitive to the reduction in soil microbial diversity, such as nitrifiers, decomposer, and denitrifiers (Griffiths et al., 2001; Wertz et al., 2006). It was speculated that through the potential functional compensation (degree of functional niche overlaps) of the species within the community, the microbial communities could still maintain the relative stable functional structure (Rosenfeld, 2002). For a microbial community to survive under environmental changes such as temperature perturbation, pinning down the possible community members that play a role in functional robustness seems like a rational strategy. Ecosystems with higher levels of biodiversity are also more stable in structure and function (Micel et al., 2003; Awasthi et al., 2014). Thus, it is natural that large-scale campus, as a semi-urban community, have a certain degree structural and functional robustness to resist sudden extreme weather changes.

Along with the time-series and partitioning sampling adopted in this work though we have utilized 16S rRNA for functional prediction of campus microbial samples, we understand that functional analysis based on 16S rRNA using neither PICRUSt nor Tax4Fun would not be of high accuracy, and more accurate functional profiling could be obtained in the future with better functional annotation database and tools. Besides, as a richer set of physicochemical data regarding air and ground surface could supplement the current patterns found in our work, future works to integrate more of such physicochemical would undoubtedly advance our understanding of campus outdoor microbial community.

## Conclusion

As the first attempt to monitor campus outdoor microbiome on spatial–temporal scale, we have obtained a unique profile of campus outdoor microbial community and presented a broad investigation of its interactions with surrounding environments. Several questions about campus outdoor microbial community have been answered in this study: first, the campus microbial community has exerted seasonality; second, temperature has larger influences than population density on campus microbial communities; and third, the functional profiles of campus microbial communities remain robust against environmental stresses. However, more of the questions are yet to come with the expectation of new and improved tools. For example, what mechanisms control these unique signature of campus outdoor microbial community? And how can mapping the campus outdoor microbial communities to guide campus planning and design? Furthermore, since university students have already been exposed to environmental microbiome as well as particulate matter and gaseous co-pollutant (Ashok et al., 2013), determination of their surrounding micro-environment should not be underestimated, if we are to fully understand the interplay of microbial and macro-environments on campus and avoid their potential threats on human health. Monitoring and protection of campus ecology (Bodelier, 2011) with microbial indicators (Sumampouw, 2014) of environmental pathogens, pollution, or degradation could be the kinds of applications toward this end.

## Materials and Methods

### Sampling Sites and Sample Processes

For investigating the microbial communities’ characteristic differences among variable seasons and sampling sites in campus, samples were collected from December 20, 2015 to April 15, 2017 in HUST campus in Wuhan, China across six seasons. Wuhan was selected because it is one of the cities with the largest number of college students in 2013 Statistic Report of National Economy, and Social Development in Wuhan (2014)), and HUST was selected for its high human density with over 50,000 students, over 3,000 staffs and 72% greenery coverage on the area of 1,153 acres (Huazhong University of Science and Technology, 2017). Different types of sampling sites were marked in distinct icons on the map (Figure 1A). We cataloged all the samples sites into three groups according to the interaction with human activities: first, samples from Classroom, Canteen, Dorm, and Library were classified “CCDL” which routinely accommodates a densely populated flow from the east to the west of the campus. Second, samples from Bus Stations, Sports Fields, Clinics, Gates, and Hotels were typical of non-routine mobility of students in campus. Third, samples from Hills and Lake could be considered as control samples with very little impact from human activities. In normal condition, three replicates were collected per specific time and site, once every other day. Scrubbing all the trails of the human beings, microbes, and other organisms, an unprecedented downpour lasted for a week in the summer of 2016, therefore, we only collected one replicate of samples before and after the downpour, separately. Besides, the Sports Field of the west campus was under repairing throughout the autumn of 2016. Finally, we obtained 251 samples from HUST with additional 17 samples collected after the summer downpour in 2016.

Moreover, 12 samples from WHU campus, 10 from CCNU campus, and 11 from HZAU campus were obtained with the same method used in HUST (refers to the Supplementary Information, sample number information refers to Supplementary Dataset 6), in the autumn of 2016 (September 23), and in the spring of 2017 (April 15).

### DNA Extraction and 16S rRNA Gene Sequencing

To obtain high-molecular-weight metagenomic DNA, a modified cetyl trimethylammonium bromide (CTAB) method was chosen (Porebski et al., 1997; Cheng et al., 2014a,b). Sterile swabs pre-moistened with 0.15 M saline solution was applied to collect samples and these swabs were cut into small strips with sterilized surgical scissors, and mixed with 5 ml lysis buffer (cetyl trimethyl ammonium bromide, 1% w/v; EDTA, 100 mM; NaCl, 1.5 mol/l; sodium phosphate, 100 mmol/l; Tris–Cl pH 8.0, 100 mmol/l) for lysis of the microbes in 50 ml centrifuge tube. Twenty microliters of Proteinase K was added to the reaction mixture, followed by gentle shaking at 100 rev/min SDS was added to a final concentration of 1%, and the reaction was incubated at 65°C for 30 min with intermittent shaking. After above steps, an equal volume of saturated phenol, chloroform, and isoamyl alcohol (25:24:1) was added and the mixture was centrifuged at 12,000 rev/min (12,114 × *g*) for 10 min to collect the supernatant that was free from protein. This procedure was repeated twice. Metagenomic DNA was precipitated with 0.6 volumes of isopropanol for 30 min at -20°C and pelleted by centrifugation at 12,000 rev/min (12,114 × *g*) for 10 min. DNA was washed twice with 70% ethanol and finally dissolved into a 200 μl of TE (1×), pH 8.0.

Before sequencing with Illumina MiSeq PE300 platform, DNA were quantified using a Qubit^®^ 2.0 Fluorometer (Invitrogen, Carlsbad, CA, United States) and their qualities were checked on a 0.8% agarose gel. To amplify the V4–V5 variable region of 16S rRNA for each individual sample with “5′-GTGYCAGCMGCCGCGGTAA-3′” as the forward primer and “5′-CTTGTGCGGKCCCCCGYCAATTC-3′” the reverse primer (Zhu et al., 2017), 5–50 ng metagenomic DNA in high quality was used as the template. DNA library for sequencing was constructed using a MetaVxTM Library Preparation Kit (GENEWIZ, Inc., South Plainfield, NJ, United States). Then, indexed adapters were added to the ends of 16S rDNA amplicons by limited-cycle PCR. And the products of limited-cycle PCR were verified by Agilent 2100 Bioanalyzer (Agilent Technologies, Palo Alto, CA, United States), and quantified by Qubit^®^ 2.0 Fluorometer (Invitrogen, Carlsbad, CA, United States). By 2 × 300 paired-end (PE) sequencing technology, the amplicons were sequenced on the Illumina MiSeq platform.

### Quality Control, OTU Clustering, and Taxonomy Assignment

To acquire high-quality taxonomical results, mothur (version 1.38.1) (Schloss et al., 2009) was used to make quality control and QIIME (V1.9.1) (Caporaso et al., 2010a) was used to do taxonomical analysis. First, PE reads were splicing with “make.contigs” command in the mothur with default settings. Afterward, all reads with the length longer than 500 bp and shorter than 300 bp, or containing ambiguous base calls (N), were removed. Then “chimrea.uchime” was applied to identify putative chimeras with the SILVA database (Quast et al., 2014) as reference, followed by the removal of putative chimeras with “remove.seqs” command in the mothur. After that, high-quality sequences were aligned by PyNAST (Caporaso et al., 2010a) and clustered into unique representative sequences by UCLUST in QIIME. Then, the Greengenes database (version 13_8) (Desantis et al., 2006) was used as the reference database for OTU classification (97% nucleotide identity). To remove singletons OTUs, the minimum reads per OTU threshold was set as 2 (Qin et al., 2010).

### Microbial Diversity Assessment

Microbial alpha-diversity and beta-diversity analysis were conducted using the QIIME (Caporaso et al., 2010b) pipeline. For alpha-diversity, rarefaction curves were drawn based on the richness metrics and evenness metrics (Solow and Polasky, 1994). For the beta-diversity analysis, Euclidean distance (the Supplementary Information) and Weighted and Unweighted UniFrac distance matrix (Lozupone and Knight, 2005) were used to measure community similarity between samples. Microbial community clustering was arrayed by PCoA and visualized by Emperor (Vázquezbaeza et al., 2013) in QIIME. The hierarchical clustering method was applied to cluster all samples. Pheatmap (pheatmap package^1^) was used to visualize Spearman correlation between different samples. PERMANOVA test (the Supplementary Information) was applied to assess the taxonomical and functional compositional difference between different groups. To fully analyze the relationship between different factors and microbial community, Mantel’s test (Mantel, 1967) was used to calculate ES and Circos (Krzywinski et al., 2009) was used for visualization.

More details about methods for statistical analyses for taxonomical and functional compositional difference assessments were provided in the Supplementary Information.

### Co-occurrence Network Analysis

Operational taxonomic units that exist in all samples, with the average RA in top 150 were selected. Based on the RA of these OTUs, Spearman correlation similarity matrix were calculated (Chaffron et al., 2010; Zhou et al., 2010; Barberán et al., 2011; Li et al., 2015). The cutoff of Spearman correlation coefficient value was set at 0.6 and the filtered co-occurrence matrix was visualized by Cytoscape (version 3.5.1) (Shannon et al., 2003). ClusterViz app (Bader and Hogue, 2003) with EAGLE algorithm in default parameters was used to identify the cluster in the network. Network nodes and edges represented OTUs and mutual exclusion relationship between OTUs, respectively.

## Author Contributions

KN designed this study. CC and HZ collected the samples. QY, HZ, and CC extracted the genomic DNA. CC, RH, ZC, MH, PY, CZ, and YZ analyzed the data. CC, ZC, and KN wrote the initial draft of the manuscript. All authors revised the manuscript.

## Conflict of Interest Statement

The authors declare that the research was conducted in the absence of any commercial or financial relationships that could be construed as a potential conflict of interest.

## Footnotes

1 http://CRAN.Rproject.org/package=pheatmapcpesnm
