# Supplementary material for: The Seasonal Dynamics and the Influence of Human Activities on Campus Outdoor Microbial Communities
Source: Front Microbiol. 2019 Jul 10;10:1579. doi: 10.3389/fmicb.2019.01579 (PMC6636667; doi:10.3389/fmicb.2019.01579)
Supplement: Supplementary file 1 [file Data_Sheet_1.PDF]

Supplementary Information

Content

Methods **2**
Sample collection 2
Statistics methods 2
Euclidean distance calculation 2
Sine curve fitting and deviation calculation 2
Mantel test 2
Permutation ANOVA 3
Function prediction 3
Functional composition and stability 3
Biomarker analysis 4
Supplementary Results **5**
PERMANOVA test results 5
Sine curve fitting and deviation calculation results 7
Supplementary Figures **8**
Supplementary Figure 1. Rarefaction curve of the sequences in all the 284 samples 8
Supplementary Figure 2. Correlation of temperature and pressure with campus microbial composition 9
Supplementary Figure 3. Correlation of UV and humidity with campus microbial composition 10
Supplementary Figure 4. Selected biomarker for each season 11
Supplementary Figure 5. Heatmaps of sample categorizations by different approaches 12
Supplementary Figure 6. General Profile of campus microbial RA at genus Level 13
Supplementary Figure 7. General profile of campus microbial RA at phylum Level 14
References **15**

Methods

Sample collection

All the samples were collected from 55 sample sites (**Figure 1(B)**, **Supplementary dataset 1)**: Classroom, Canteen, Dorm, Library, Gate, Lake, Clinic, Hotel, Bus Station, Hill, Sports Field in HUST; Classroom, Canteen, Dorm, Library, Gate, Lake, Clinic, Sports Field in WHU; Classroom, Canteen, Dorm, Library, Gate, Clinic, Sports Field in CCNU; Classroom, Canteen, Dorm, Library, Gate, Lake, Clinic, Sports Field in HAU (**Supplementary dataset 2**). Except for the bad weather, sample collections were implemented every other day for three times during consecutive sunny days for each season to eliminate unexpected deviation. To ensure sufficient replicates and plenty of bacterial quantity acquired from the habitat areas, 5 sterile swabs pre-moistened with 0.15M saline solution was applied to scratch against the surface of 5 parts (approximate 20 cm^2) in the vicinity for each sampling site (2 m^2) in serpentine way. Then the 5 swabs from the same sites were put together into a sterile tube and stored at -80 °C for subsequent treatment.

Statistical methods

Euclidean distance calculation

For calculating the similarities of the matrix (Supplementary dataset 3, Supplementary dataset 4) used for sample similarities test (Figure 3 (G) (I), Figure 4 (B)) and in this study, we used Euclidean distance. R script 'dist' function was applied to generate the Euclidean distance (refers to the formula (1)). Imported file was the average abundance table of microbial community in each season at genus level. Method parameter was set as 'eulidean'. The relative abundances of microbial relative abundance table of microbial community in 2015 winter served as the reference.

$$\text{Distance}(p, q) = \sqrt{\sum_{i}^{n} (qi - pi)^2} \qquad (1)$$

Where in Distance(p, q) (1), pi represented the average RA of the *ith* genus in the group p and qi represented the average RA of the *ith* genus in the group q. The sum of relative abundance of the taxa in each group is an accumulating features. n represented the total number of genuses in the microbial community of all the samples.

Sine curve fitting and deviation calculation

Scatters indicated the Euclidean distance (for sample similarity) and the corresponding temperature for each season. Sine functions for two groups of data were calculated. Seasons were numbered from 1~6 in correspond to the winter of 2015, spring, summer, autumn, winter of 2016, spring of 2017. Simulated fitting lines consisted of linear part and cyclical part, the former one indicated the deviation of the curve among different years and the latter one revealed the seasonality of the sample similarity and temperature variations between seasons.

Mantel test

To assess the effect size of potential drivers on the microbial structure and function (**Figure 6**), Mantel test was conducted on R command 'mantel' in library 'vegan'(1) based on Euclidean distance. The test data was transformed into a 2-dimension matrix, with each column representing the detected values for each variable, in this context, the OTUs at genus level, and each row presenting the detection of relative abundance of all the OTUs in each sample. The 'environment' of the test was also input as a 2-dimension matrix with the same row title and each column presenting different grouping information. While running the script, the number of permutation was set to 9999.

Permutational multivariate ANOVA

Depicting the differences among groups (**Figure 3(A-F)**, **Figure 4(A-C)**, **Figure 5(A-K)**), PERMANOVA test was run on R command 'adonis' in library 'vegan'. The test data was transformed into a 2-dimension matrix, with each column presenting the detected values for each variable and each row presenting each detection of each sample. The 'environment' of the test was also input as a 2-dimension matrix with the same row title and each column presenting different grouping information. While running the script, the column name was assigned as 'environment' and method was assigned as 'Bray' (refers to Bray-Curtis).

Function predication

Predictive functional profiling of campus microbial communities was generated by PICRUSt 1.1.0 from 16S rRNA marker genes(2), which allows the prediction of functional pathways from the 16S rRNA reads. First, a collection of closed-reference OTUs was obtained by using QIIME 'pick_closed_reference_otus.py' script in default settings. Then the script 'normalize_by_copy_number.py' was applied to normalize the output table based on the predicted 16S rRNA copy number. Final functional predictions, inferred from the metagenomes, were generated with the script 'predict_metagenomes.py'.

As a database that maps prokaryotic clades to established metabolic or other ecologically relevant functions based on culturable strains currently reported on literatures, Functional Annotation of Prokaryotic Taxa (FAPROTAX) was also used for providing further clues to the hypothesis on functional stability. The input data was the same as those used for functional profiling by PICRUSt， formed by QIIME with 'pick_closed_reference_otus.py' script, but analyzed by FAPROTAX1.1(3) with "collapse_table.py" scripts in default settings. And the predicted functional profiles by FAPROTAX was referred to in **Supplementary dataset 7.**

Functional composition and stability

The predicted functions (based on PICRUSt annotations) and their relative abundances (based on the reads assigned to certain functions) for the microbial community was defined as the functional composition of the microbial community. And the definition of functional stability: Plotting all samples onto PCoA scale, each based on the distribution of relative abundances of functions for the microbial community examined. Then PERMANOVA test is used to evaluate if samples are

clustered together, if not statistically, then they are considered not stable, otherwise stable. The PERMANOVA test results indicate the functional stability quantitatively.

Biomarker analysis

Linear discriminate analysis (LDA) effect size (LEfSe)(4) was used to screen the biomarkers that acted as typical microbial signatures for each season (**Supplementary Figure 4**). Specifically, tables with the group information and the taxa abundance at taxonomical level (**Supplementary dataset 4**) were imported into the LEfSe pipeline and the parameters were set as follows: the alpha value for the factorial Kruskal-Wallis test between classes and the p-value for the pairwise Wilcoxon test between subclass were both 0.05. For discriminating the features, the threshold for the logarithmic LDA score was 2.0 by default.

Supplementary Results

PERMANOVA test results

All the results for PERMANOVA test were shown in the **Supplementary Table 1** and **Supplementary Table 2**.

Supplementary Table 1. Single factor PERMANOVA test. Results of single factor PERMANOVA test in functional and taxonomical level. ‘R^2’ indicates the percentage of variation explained by each factor, ‘Pr’ value represents the significance of each factor, the ‘Group’ column represents the factors and test data level (functional or taxonomical). Here, we used labels without suffix, e.g., "Seasons, taxonomical", to represent the association of taxonomical structure with environmental factors; and used labels with suffix "-PICRUSt", e.g., "Seasons, functional-PICRUSt", to represent the association of functional structure with environmental factors by three different methods: PICRUSt, FAPROTAX and Tax4Fun. For the significance test of the influence of grade and sex to the microbial community from the Dorms, the samples collected from winter and spring were used for PERMANOVA test.

Group	R2	Pr(>F)	F
Season, taxonomical	0.120219856	0.0001	11.25065343
Season, functional-PICRUSt	0.095923	0.0004	8.735605823
Season, functional-FAPROTAX	0.074602495	0.0001	6.63744185
Season, functional-Tax4Fun	0.089038845	0.0001	8.047395727
CCDL & Hills & Lakes, taxonomical	0.121568225	0.0001	9.272286458
CCDL & Hills & Lakes, functional-PICRUSt	0.020374848	0.2116	1.393507316
CCDL & Hills & Lakes, functional-FAPROTAX	0.044067644	0.0383	3.088641262
CCDL & Hills & Lakes, functional-Tax4Fun	0.147267711	0.0001	11.57096633
CCDL & Others, taxonomical	0.019451383	0.0001	4.939473982
CCDL & Others, functional-PICRUSt	0.003671712	0.3594	0.917625487
CCDL & Others, functional-FAPROTAX	0.008168406	0.0851	2.050683909
CCDL & Others, functional-Tax4Fun	0.016421799	0.0023	4.157298316
CCDLE & CCDLMW, taxonomical	0.03073941	0.211	1.268571
CCDLE & CCDLMW, functional-PICRUSt	0.02195	0.357	1.0662
Hotels & Others, taxonomical	0.01281078	0.0075	3.231279465
Hotels & Others, functional-PICRUSt	0.001757974	0.5981	0.438506307
Hotels & Others, functional-FAPROTAX	0.001002134	0.9012	0.249781677
Hotels & Others, functional-Tax4Fun	0.004343686	0.3387	1.086296429
Gates & Others, taxonomical	0.016671056	0.0018	4.221469373
Gates & Others, functional-PICRUSt	0.005894228	0.1873	1.476364759
Gates & Others, functional-FAPROTAX	0.003760866	0.3556	0.939990699
Gates & Others, functional-Tax4Fun	0.015521449	0.0044	3.92577441
CCDL & Hotels, taxonomical	0.032033585	0.0022	3.938149609
CCDL & Hotels, functional-PICRUSt	0.003540428	0.6265	0.422807831
CCDL & Hotels, functional-FAPROTAX	0.001967776	0.8718	0.234627005

CCDL & Hotels, functional-Tax4Fun	0.015997007	0.0958	1.934591469
CCDL & Gates, taxonomical	0.043124259	0.0002	5.4982684
CCDL & Gates, functional-PICRUSt	0.007889082	0.3295	0.970121429
CCDL & Gates, functional-FAPROTAX	0.002794121	0.7703	0.341837908
CCDL & Gates, functional-Tax4Fun	0.038595123	0.0014	4.897629632
Campus, taxonomical	0.025336364	0.0032	2.426198331
Campus, functional-PICRUSt	0.00794821	0.4679	0.747776439
Campus, functional-FAPROTAX	0.009075315	0.4195	0.85478685
Campus, functional-Tax4Fun	0.016941745	0.0763	1.60847999
Canteens & Others, taxonomical	0.008202266	0.0474	2.059254743
Canteens & Others, functional-PICRUSt	0.004127316	0.2958	1.031960866
Canteens & Others, functional-FAPROTAX	0.011865418	0.0508	2.989966332
Canteens & Others, functional-Tax4Fun	0.005381213	0.2194	1.347171392
Classrooms & Others, taxonomical	0.005681484	0.1611	1.422772856
Classrooms & Others, functional-PICRUSt	0.004161304	0.2924	1.040494628
Classrooms & Others, functional-FAPROTAX	0.00414006	0.2931	1.035160669
Classrooms & Others, functional-Tax4Fun	0.022757334	0.0003	5.798535264
Dorms & Others, taxonomical	0.017626727	0.0008	4.467807827
Dorms & Others, functional-PICRUSt	0.008720053	0.1039	2.190393515
Dorms & Others, functional-FAPROTAX	0.004437262	0.3024	1.109802719
Dorms & Others, functional-Tax4Fun	0.014440839	0.0058	3.648455592
Libraries & Others, taxonomical	0.007397163	0.0664	1.855619957
Libraries & Others, functional-PICRUSt	0.004788003	0.2623	1.197948584
Libraries & Others, functional-FAPROTAX	0.005047015	0.235	1.263081632
Libraries & Others, functional-Tax4Fun	0.012272996	0.0128	3.093948051
Lakes & Others, taxonomical	0.032066162	0.0001	8.248987755
Lakes & Others, functional-PICRUSt	0.004486687	0.2675	1.122220158
Lakes & Others, functional-FAPROTAX	0.027375158	0.0022	7.008266726
Lakes & Others, functional-Tax4Fun	0.029171966	0.0001	7.482086652
Sports fields & Others, taxonomical	0.133744613	0.0001	38.44410004
Sports fields & Others, functional-PICRUSt	0.015069889	0.0372	3.809815784
Sports fields & Others, functional-FAPROTAX	0.085634403	0.0001	23.31995689
Sports fields & Others, functional-Tax4Fun	0.030120531	0.0001	7.732932248
Stations & Others, taxonomical	0.012019246	0.0093	3.029200858
Stations & Others, functional-PICRUSt	0.004590727	0.2698	1.148362785
Stations & Others, functional-FAPROTAX	0.005176008	0.2358	1.295531699
Stations & Others, functional-Tax4Fun	0.017645892	0.0023	4.472752911
Clinics & Others, taxonomical	0.013160219	0.0089	3.320594264
Clinics & Others, functional-PICRUSt	0.001395966	0.6559	0.348081534
Clinics & Others, functional-FAPROTAX	0.001927515	0.6308	0.48087825
Clinics & Others, functional-Tax4Fun	0.011048727	0.0224	2.781869095
Hills & Others, taxonomical	0.022078507	0.001	5.621666369
Hills & Others, functional-PICRUSt	0.006894558	0.1406	1.728663393
Hills & Others, functional-FAPROTAX	0.003798656	0.2866	0.949471963

Hills & Others, functional-Tax4Fun	0.058146047	0.0001	15.37219827
Grade, taxonomical	0.035967407	0.3238	1.119279796
Grade, functional-PICRUSt	0.007924912	0.8563	0.239646549
Grade, functional-FAPROTAX	0.00770891	0.9007	0.233063955
Grade, functional-Tax4Fun	0.03963775	0.2797	1.238212451
Gender, taxonomical	0.092161124	0.0025	3.045511494
Gender, functional-PICRUSt	0.080096259	0.082	2.612107833
Gender, functional-FAPROTAX	0.085745671	0.0417	2.813626416
Gender, functional-Tax4Fun	0.060553921	0.1102	1.933711426

Supplementary Table 2. Dual factor PERMANOVA test. Results of dual factor PERMANOVA test.in functional and taxonomical level. 'R^2' indicates the percentage of variation explained by each factor, 'Pr' value represents the significance of each factor, the 'Group' column represents the test level (Taxonomical or functional).

Group	Human density	Season
Taxonomical	R^2=0.04879,<br>Pr (>F) =0.001,<br>F=9.0654	R^2=0.19913,<br>Pr (>F) =0.001,<br>F= 14.8006
Functional	R^2=0.00504,<br>Pr (>F) = 0.403,<br>F=0.9136	R^2= 0.16934,<br>Pr (>F) =0.001,<br>F= 12.2859

Sine curve fitting and deviation calculation results

Scatters indicated the Euclidean distance (for sample similarity) and the corresponding temperature for each season (**Supplementary Table 3**). Sine functions for two groups of data were calculated. Seasons were numbered from 1~6 in correspond to the winter of 2015, spring, summer, autumn, winter of 2016, spring of 2017. Simulated fitting lines consisted of linear part and cyclical part, the former one indicated the deviation of the curve among different years and the latter one revealed the seasonality of the sample similarity and temperature variations between seasons.

Supplementary Table 3. Sine curve fitting formula. Y_{SD1} and Y_{SD2} were used for fitting the sample distance variations and Y_{T1} and Y_{T2} used for fitting the temperature variations.

Data	Linear part	Cyclical part
**Sample distance**	Y_{SD1}=-0.003X+0.003	Y_{SD2}=0.027sin(1.57X-3.14)+0.012
**Temperature**	Y_{T1}=0.1X+10.32	Y_{T2}=13.91sin(1.57X-3.14)+11.46

Supplementary Figures

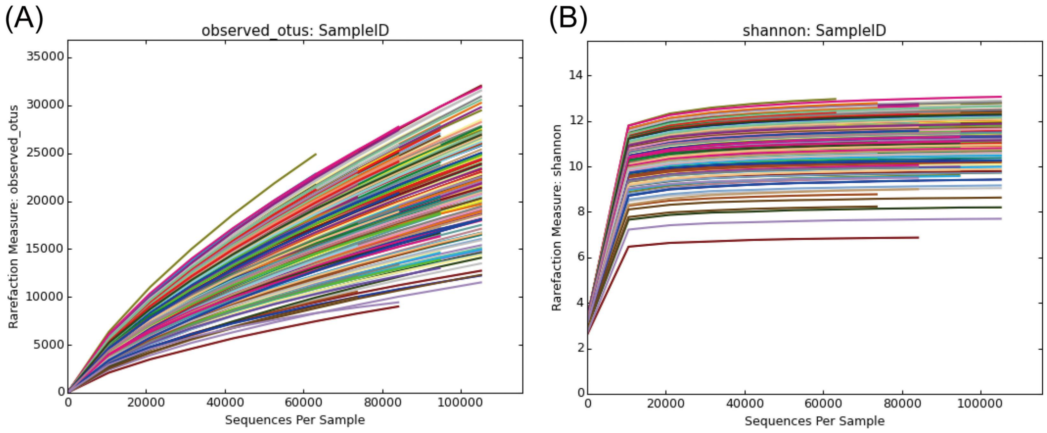

Supplementary Figure 1. Rarefaction curve of the sequences in all the 284 samples. Rarefaction measurement of observed **(A)** OTUs and **(B)** Shannon-index versus number of sequences per sample.

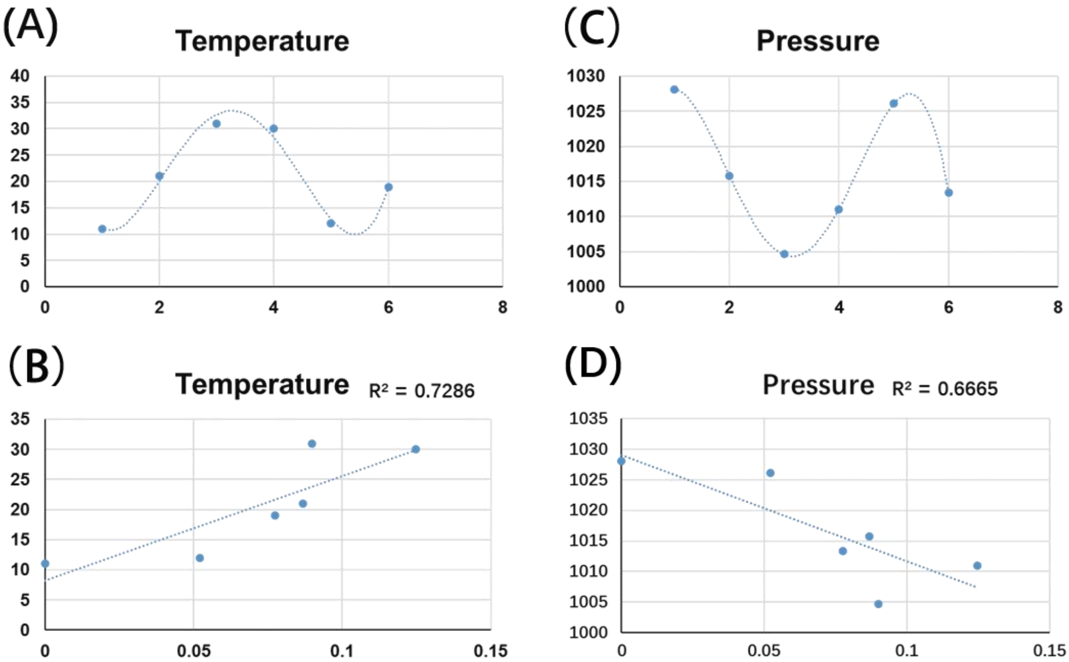

Supplementary Figure 2. Correlation of temperature and pressure with campus microbial taxonomical composition. The provenance of average temperature and pressure in Wuhan was obtained from the World Weather Online(5). The fitting curve was applied to simulate the variation trend of **(A)** temperature and **(B)** pressure. And the correlations of sample similarity with **(C)** temperature and **(D)** barometric pressure were evaluated by the linear regression algorithm.

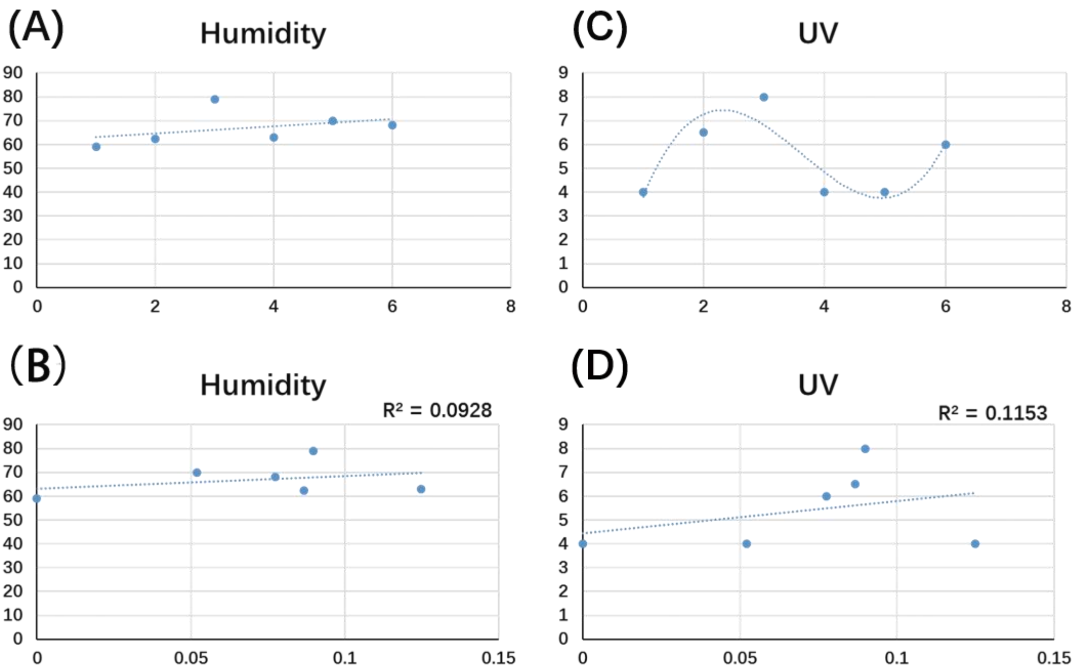

Supplementary Figure 3. Correlation of UV and humidity with campus microbial taxonomical composition. The data provenance and evaluation methods were similar to those of Supplementary Figure 4. The fitting curve was applied to simulate the variation trend of **(A)** humidity and **(B)** UV. And the correlations of sample similarity with **(C)** humidity and **(D)** UV were evaluated by the linear regression algorithm.

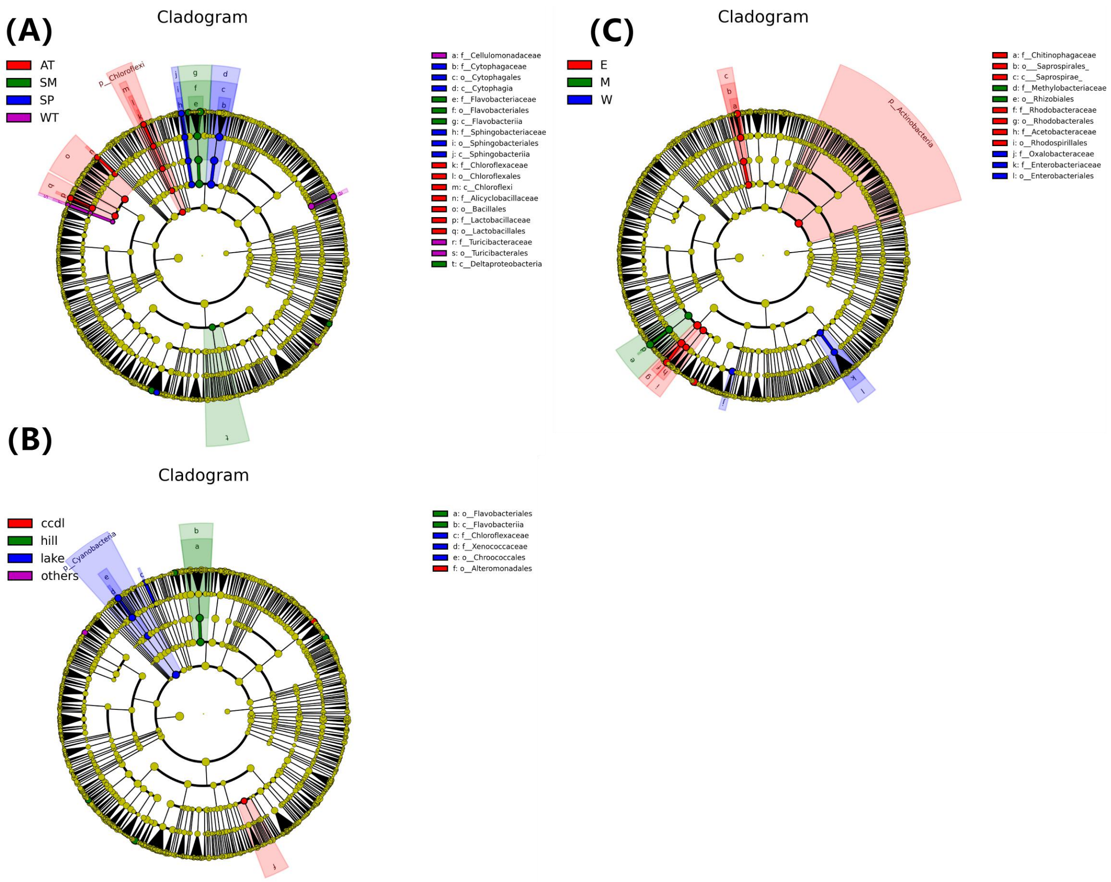

Supplementary Figure 4. Selected biomarkers for each season. Cladograms were generated based on different grouping approaches by LEfSe to visualize biomarkers at the genus level. **(A)** For season**,** WT indicated winter, SP for spring, SM for summer, while AT indicated autumn. **(B)** Samples were categorized according to human density: CCDL (classroom, canteen, dorm and library), hills, Lakes and others. **(C)** For geographic factors, E indicated sampling sites in the east of the campus, M for the middle of campus and W for the west of the campus. Typical biomarkers for each group were shaded in different colors.

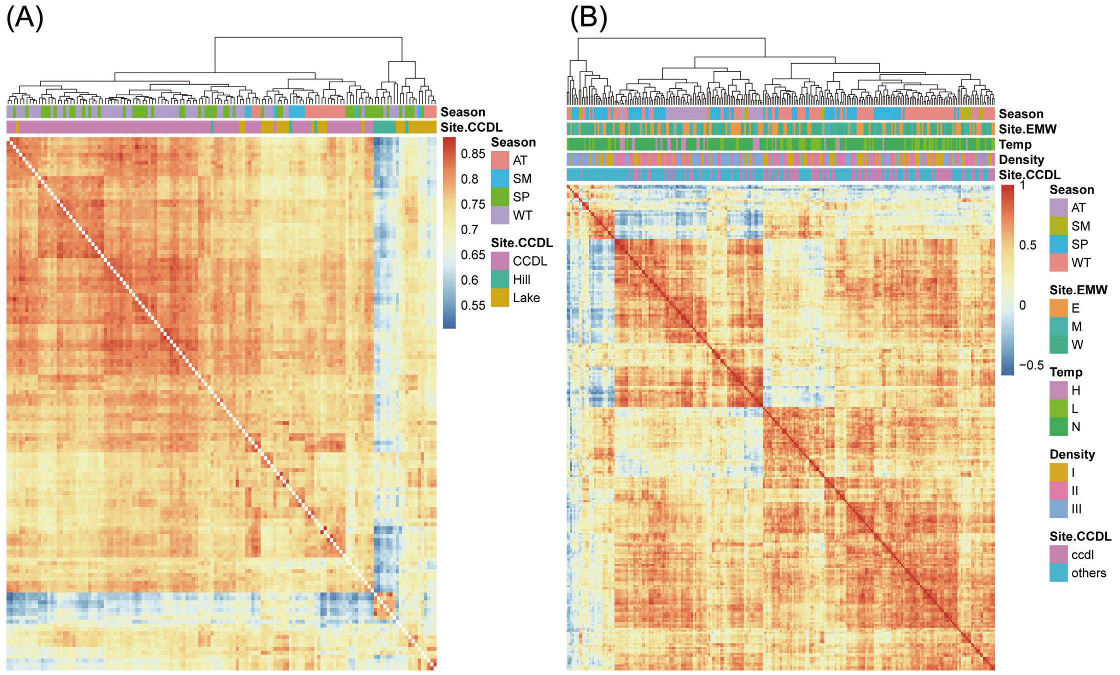

Supplementary Figure 5. Heatmaps of sample categorizations by different approaches. The matrix of relative abundances of assigned OTUs in all 284 samples were depicted and categorized in heatmap. Each column depicted different samples arrayed by hierarchical clustering. The upper bars containing different colors indicated the different categorizations for comparison. **(A)** The categorization of season and site. CCDL were drawn for comparison. For site. CCDL, the range of sampling sites was curtailed to CCDL, hills and lakes. For season: AT indicated autumn, SM for summer, SP for spring and WT for winter. **(B)** For site. EMW, E represented sampling sites in the east of the campus, M represented the sampling sites in the middle of the campus while W represented those in the west of the campus; for Temp, temperature in each sampling site were divided into high (T > 37 °C), low (< 10 °C) and normal (10 °C < T < 37 °C); for density, sampling sites in group I featured the highest human density including classroom, canteen, dorm and library, those in group II featured lower human density including bus stations, sports fields, clinics, gates and hotels, while those in group III featured lowest human density including hills and lakes; for site. CCDL all sampling sites were categorized into two groups, classroom, canteen, dorm and library were in 'CCDL', while all other sites were defined as 'others'.

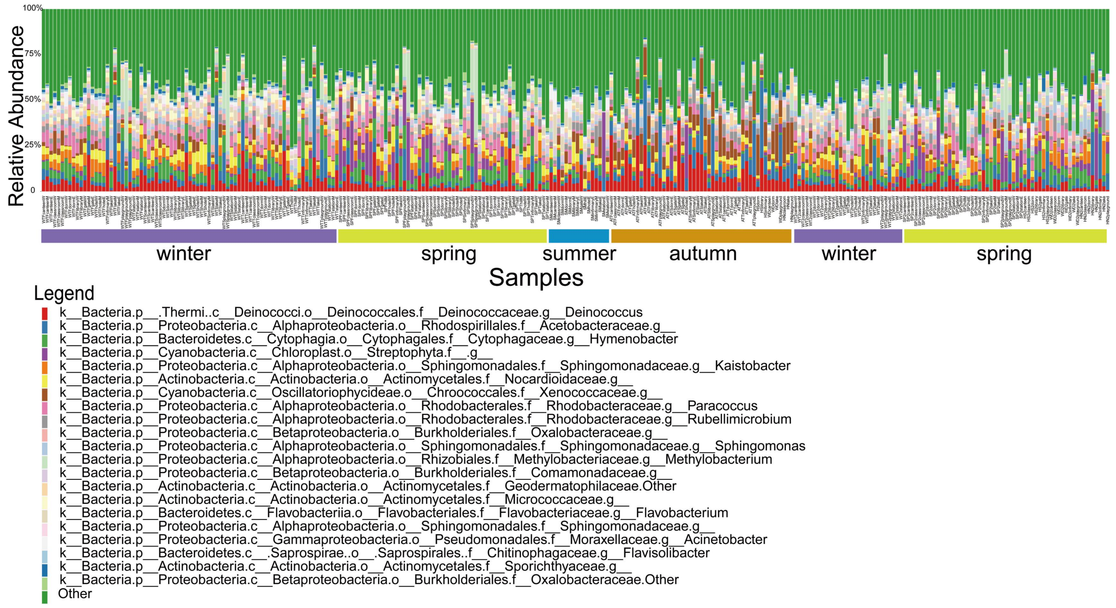

Supplementary Figure 6. General Profile of campus microbial RA at genus Level. The samples were collected from 55 different sites over 6 seasons from the winter of 2015 to the spring of 2016. Relative abundance (RA) of top 21 classes were depicted by different colors (specific details were shown in the legend) while taxons with lower RA were classified as 'other'.

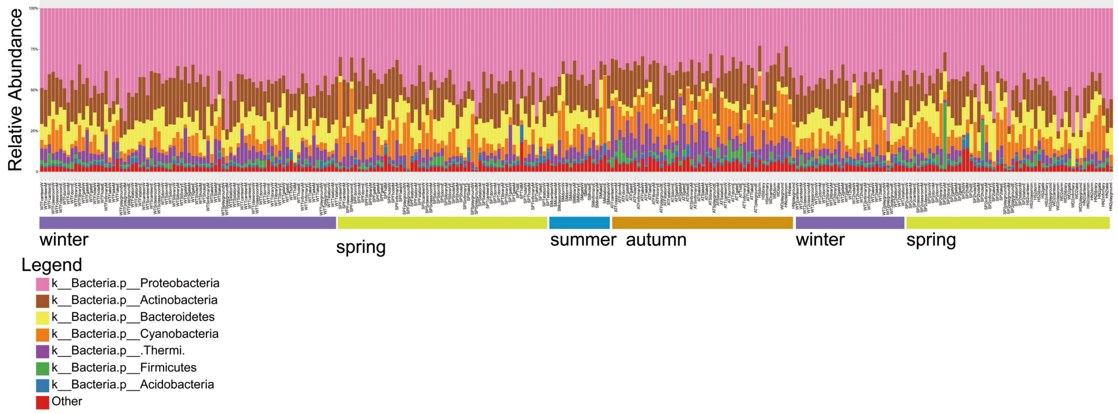

Supplementary Figure 7. General profile of campus microbial RA at phylum Level. The samples were collected from 55 different sites over 6 seasons from the winter of 2015 to the spring of 2016. Relative abundance (RA) of top 7 classes were depicted by different colors (specific details were shown in the legend) while taxons with lower RA were classified as ‘other’.

References

1. Jari Oksanen FGB, Michael Friendly, Roeland Kindt, Pierre Legendre, Dan McGlinn, Peter R. Minchin, R. B. O'Hara, Gavin L. Simpson, Peter Solymos, M. Henry H. Stevens, Eduard Szoecs, Helene Wagner. vegan: Community Ecology Package. (2017).

2. Langille MG, Zaneveld J, Caporaso JG, McDonald D, Knights D, Reyes JA, et al. Predictive functional profiling of microbial communities using 16S rRNA marker gene sequences. *Nature Biotechnology* (2013) 31(9):814-21. doi: 10.1038/nbt.2676. PubMed PMID: 23975157; PubMed Central PMCID: PMCPMC3819121.

3. Louca S, Parfrey LW, Doebeli M. Decoupling function and taxonomy in the global ocean microbiome. *Science* (2016) 353(6305):1272-7.

4. Segata N, Izard J, Waldron L, Gevers D, Miropolsky L, Garrett WS, et al. Metagenomic biomarker discovery and explanation. *Genome biology* (2011) 12(6):R60. doi: 10.1186/gb-2011-12-6-r60. PubMed PMID: 21702898; PubMed Central PMCID: PMC3218848.

5. World Weather Oline： https://www.worldweatheronline.com (2017, November).
